# Microfluidic and Paper-Based Devices for Disease Detection and Diagnostic Research

**DOI:** 10.3390/ijms19092731

**Published:** 2018-09-12

**Authors:** Joshua M. Campbell, Joseph B. Balhoff, Grant M. Landwehr, Sharif M. Rahman, Manibarathi Vaithiyanathan, Adam T. Melvin

**Affiliations:** Cain Department of Chemical Engineering, Louisiana State University, Baton Rouge, LA 70803, USA; jcamp62@lsu.edu (J.M.C.); jbalho1@lsu.edu (J.B.B.); glandw1@lsu.edu (G.M.L.); srahm12@lsu.edu (S.M.R.); mvaith1@lsu.edu (M.V.)

**Keywords:** microfluidic devices, µPADs, lateral flow strip assays, LFSAs, single cell analysis, high-throughput screening, point of care, polydimethylsiloxane, PDMS

## Abstract

Recent developments in microfluidic devices, nanoparticle chemistry, fluorescent microscopy, and biochemical techniques such as genetic identification and antibody capture have provided easier and more sensitive platforms for detecting and diagnosing diseases as well as providing new fundamental insight into disease progression. These advancements have led to the development of new technology and assays capable of easy and early detection of pathogenicity as well as the enhancement of the drug discovery and development pipeline. While some studies have focused on treatment, many of these technologies have found initial success in laboratories as a precursor for clinical applications. This review highlights the current and future progress of microfluidic techniques geared toward the timely and inexpensive diagnosis of disease including technologies aimed at high-throughput single cell analysis for drug development. It also summarizes novel microfluidic approaches to characterize fundamental cellular behavior and heterogeneity.

## 1. Introduction

Clinicians and patients require fast, affordable, and easy-to-use methods to detect and diagnose diseases in order to facilitate timely and effective treatment to reach the best possible medical outcome. For example, over 5 million people around the world die annually from sepsis [1] and approximately 450,000 die annually of malaria [2], but with easy-to-use and accessible devices, the mortality rate of these diseases could decrease, especially in countries without advanced healthcare. To address these issues, researchers have developed microfluidic devices in order to provide inexpensive and facile detection of diseases [3]. Moreover, these devices allow for direct and rapid interrogation of complex cells, which has allowed for researchers to obtain a more comprehensive understanding of diseases by analyzing heterogeneous cellular behavior and genetics to identify better treatment metrics for patients with higher rates of success [4]. As the utility for microfluidic devices is vast, this review is focused on two broad areas on the use of microfluidic devices: their role in disease detection and their use in diagnostic understanding. These two main sections will focus on the relationship between these two aspects of patient care by analyzing recent developments in microfluidic devices used for these purposes and discussing the future direction of the field.

The identification of human illnesses can be categorized by detecting one or more of four potential disease indicators: biomarkers, human cells, bacteria, and viruses. In some cases, the presence of specific biomarkers such as proteins, sugars, metabolites, and other compounds can provide early information about certain disorders. For example, prostate specific antigen (PSA) has garnered significant attention as a possible detector of prostate cancer [5]. With the advent of antibiotic-resistant bacteria, early detection of bacterial infections has become of increasing importance for patient treatment [6]. Finding ways to identify resistant bacteria can provide essential information to clinicians to decide upon the best treatment option, highlighting a need for devices that are capable of efficiently detecting and classifying bacteria. Similarly, the ability to remotely confirm a viral infection in low income areas or underdeveloped countries would provide essential information to slow the progression of diseases. Examples include the rapid and accurate detection of influenza [7,8,9,10], Zika virus [11,12,13], or sexually transmitted diseases [11,14,15]. To this extent, the first part of this review will highlight some recent microfluidic devices developed to provide early and accurate detection of diseases by confirming the presence or absence of specific precursors and identifiers.

Furthermore, in order to effectively treat patients, clinicians and scientists need additional information beyond the presence or absence of cells and biomolecules, which has sparked the need for microfluidic technologies to study fundamental cellular behavior during disease progression and to analyze single cells to elucidate population heterogeneity [4]. This fundamental understanding can provide new insight into personalized medicine and aid in the development of new drugs for treatment. In the past, these studies relied on bulk endpoint measurements; however, new technology has facilitated the analysis of single cells to gather more information about the heterogeneity within a disease [16]. At the forefront of this technological innovation is the use of microfluidic devices. Microfluidics allow for highly sensitive and controllable conditions by facilitating the predictable passage of fluids through microchannels due to laminar flow. This sensitivity can be easily manipulated to provide researchers with ways to produce detection and single cell analysis methods that cannot be recreated on a larger scale [17]. Polydimethylsiloxane (PDMS), the material most used in microfluidic fabrication, provides a relatively inexpensive platform for research, especially when compared to common biological assays because PDMS is widely manufactured, and these devices require a small amount of sample and reagents [18]. Additionally, these technologies are optimized to be high-throughput, capable of the rapid analysis of a large number of samples [17]. In addition to devices used for disease detection, this review discusses microfluidic technologies that have been developed to understand fundamental cellular behavior and heterogeneity, to develop drugs more efficiently and quickly, and to sequence genetic profiles of single cells. Devices aimed to increase the understanding of cell behavior and heterogeneity will characterize individual cell responses so that mutations or dysregulation of signaling networks affecting these cells can be better understood from a fundamental standpoint to provide essential information on how to diagnose and treat a disease [19]. Technologies that test the efficacy of drugs in a high throughput manner are necessary to speed up the relatively slow process of drug development and match certain compounds with their effects on different diseased cells [20]. Genetic sequencing of individual cells helps with both understanding cell behavior and drug discovery since being able to identify genetic differences can provide researchers and clinicians with another tool to diagnose and treat patients [21]. 

Therefore, while clinicians are concerned with correct and early detection of diseases within patients, researchers focus on developing methods to identify these diseases by first understanding the fundamental breakdown of the biology responsible for such diseases. One area of both detection and diagnosis is the isolation, identification, and study of circulating tumor cells (CTCs) during cancer metastasis. CTCs have garnered significant attention in recent years as markers of cancer progression and have emerged as a prominent drug target. This review will not highlight devices used to detect or study CTCs because several review articles have been recently published on this area [22,23,24,25,26].

## 2. Disease Detection

**Figure 1:**
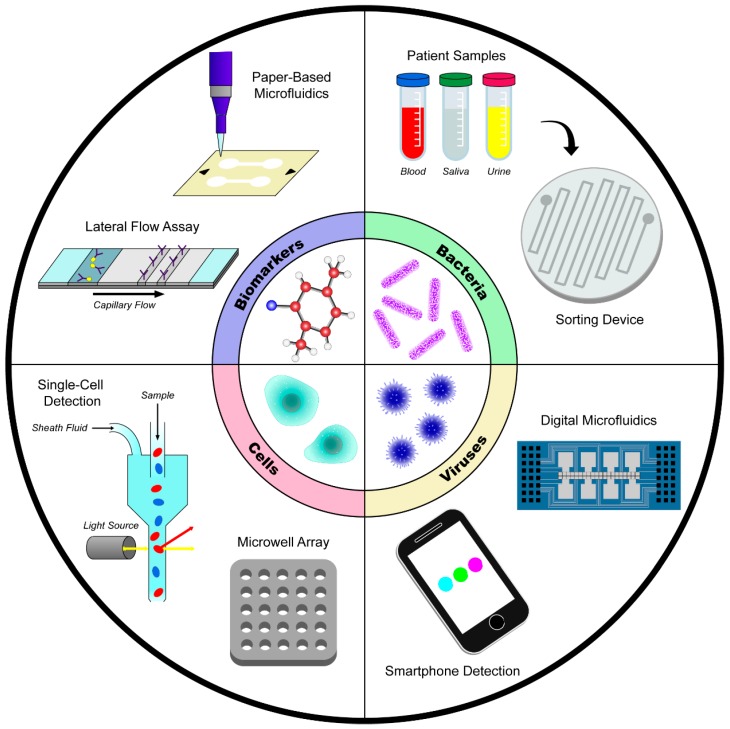
Microfluidic devices for biomarker, human cell, bacteria, and virus detection. Each panel contains some representative devices reviewed within this section.

In 2016 approximately 54.7 million deaths occurred worldwide, and, of those deaths, over 50 million were attributable to communicable and non-communicable diseases such as HIV/AIDS, malaria, tuberculosis, cancer, and chronic respiratory diseases [27]. Early detection of these diseases has the potential to reduce mortality rates, and portable, easy-to-use, and affordable devices provide an optimal platform for disease recognition, especially in underdeveloped nations. The majority of microfluidic devices that have been recently developed work by sensing specific biomarkers, human cell types, bacteria, or viruses (Figure 1). While many devices focus on one of those four areas of detection, a few devices identifies fungi such as *Candida*; however, these will not be reviewed here [28].

### 2.1. Detection of Biomolecules and Biomarkers

To facilitate the early and correct diagnosis of diseases, clinicians must be able to identify the type of infection or key biomarkers associated with a disease within the patient. The identification process includes collecting patient samples that require less invasive methods (e.g., blood, saliva, or urine) or more invasive methods (e.g., direct tissue samples). Lab technicians within a clinical setting can test these samples for pathogens or specific biomarkers such as proteins, sugars, or other biomolecules relevant to certain diseases. This section highlights new approaches using lab-on-chip devices to isolate, detect, and quantify pathogens and biomarkers to provide insight on disease progression (Table 1).

#### 2.1.1. Lateral Flow Strip Assays to Detect Biomolecules

Lateral flow strip assays (LFSAs) are an easy-to-use and portable platform for the detection of specific biomolecules. The most widely used and commercially available LFSA is the home pregnancy test, which identifies hormones in urine. This same concept can be applied for recognizing other biomarkers to diagnose a patient with a disease or illness such as the detection of myeloperoxidase as a neutrophilic bronchitis precursor [29]. These assays work by taking a liquid patient sample (e.g., blood, urine, or saliva) and applying that sample to an absorbent pad on one side of the strip. The sample travels across the strip assay, which has two lines in the middle of the device—one as the control line and one as the test line. Although the lines within these devices utilize different methods for biomolecule binding and identification, almost all LFSAs indicate a positive result with two lines (control line and test line present) and a negative result with only one line (control line present only). Most of these strip assays are very easy to use since LFSAs require small sample sizes on the order of 10–100 μL, and results can be easily interpreted by the naked eye [30]. Since LFSAs are comprised of a single strip of absorbent material with detection molecules deposited in two lines along the middle of the device, they are also simple and cheap to manufacture providing an affordable medical test that can be used either at home or in the clinic. LFSAs do not take long to show results because they are limited only by the amount of time that it takes for the sample to wick across the sample pad, with some devices taking only 5 min to produce results [30].

Many LFSAs utilize antibodies to detect specific biomolecules [29,31,32,33]. Before applying the sample to the strip, it must be mixed with detection antibodies that bind to the target molecule that is conjugated to a secondary molecule to provide an analytical output for qualitative analysis. Although there are several ways in which these results can be generated, most LFSAs consist of a control line that is composed of immobilized capture antibodies to complement the detection antibodies to ensure proper sample loading. The second line, or test line, contains immobilized antibodies that bind to the target molecule. Current LFSA results can be interpreted as a fluorescent signal present on the test line [32], as a visually present test line [29,30,33], or by measuring a pressure output from a chemical reaction [31].

A recently developed LFSA uses a combination of gold nanoflowers conjugated to antibodies and quantum dot nanobeads to produce a fluorescent signal in the presence of tetrodotoxin (TTX), a potent neurotoxin present within marine life, as a detection method for patients who have potentially consumed contaminated food or to test food sources [32]. Oliveira-Rodriguez et al. used a strip assay by conjugating antibodies to nanoparticles for the quantitative detection of extracellular vesicles [33], whereas Wolfe et al. used a similar approach to detect myeloperoxidase from sputum samples to diagnose neutrophilic bronchitis [29]. While still providing a visual result, Raston et al. developed an LFSA that incorporates aptamers conjugated to nanoparticles to bind to specific target molecules such as Vaspin as a biomarker for diabetes-related diseases [30]. Yet other strip assays do not use visual identification for reading results, instead using chemical reactions to produce gases such as hydrogen peroxide that can be measured as a change in pressure [31]. 

While many LFSAs are restricted to research labs, they exhibit great potential for clinical or portable detection of biologically relevant molecules for disease identification and progression (Table 2). Strip assays often provide good limits of detection comparable to current tests such as ELISA but at vastly reduced costs and increased ease of use. Unfortunately, some LFSAs exhibit poor limits of detection at clinically relevant concentrations and will require further optimization. While LFSAs can identify if a biomarker is present, they can be limited in their ability to provide quantifiable results which is why most LFSAs are not currently used in a clinical, but have been restricted to research labs.

#### 2.1.2. Paper Microfluidic Devices to Detect Biomolecules and Biomarkers

Microfluidic paper-based analytical devices (µPADs) are similar to LFSAs but do not rely on two lines to report results allowing for a wider range of applications. µPADs have garnered significant interest in recent years for detecting a range of biomolecules due to their low cost and ease of use. The type of paper, the geometry of the device, and the coating of the paper all allow for different applications and biological targets to be interrogated. For this review, only systems that are specifically used to detect biomolecules for disease diagnosis and progression will be discussed; however, readers are encouraged to refer to more comprehensive reviews on µPADs for more in-depth discussion [49,50]. 

An advantage of µPADs is their simple design that allows for facile operation with little to no training prior to use. In addition to their ease of use, the individual devices are inexpensive and easy to produce, making µPADs good prospects for manufacturing, and they do not require complex machinery to analyze their outputs which makes them ideal candidates for clinical use. A limitation of some µPADs is their inability to detect biologically relevant concentrations of their desired biomolecules coupled with the use of many new devices being restricted to the lab [34]. Although a few devices provide quantitative, rather than qualitative, results, these assays typically require non-ambient conditions to function correctly [35,37]. 

µPADs for biomolecule detection work by patterning the paper material with chemicals to bind to the desired molecule resulting causing a visual color change [34,35,36], or by integrating a printed electrode with detection chemicals on the paper providing an electrical or electrochemiluminescent output [37]. Some devices are composed of separate sample and results regions housed by specialized casing to facilitate testing conditions [34,35]; however, the majority of µPADS use a cloth or paper cutout for detection [36,37]. Some µPADs are designed for simultaneous, multiplexed detection of glucose, nitrates, and proteins using colorimetric readouts and paper-based actuators, allowing for fast detection of many molecules [34]. A recent device developed by Messina et al. provided a quantitative concentration of phenylalanine in phenylketonuria (PKU) patients from a urine sample using a chemical reaction resulting in a change of color intensity within a temperature-controlled housing [35]. A µPAD developed by Li et al. allowed for the detection of multiple biomolecules using paper cutouts that change color and are loaded within a micropipette tip [36]. Yao et al. developed a device that used electrodes and electrochemiluminescence to measure lactate within a saliva sample by applying directly to the tongue [37]. While µPADs have not been adopted heavily in the clinic yet, they are of great interest within the research community and may provide clinicians with inexpensive and easy-to-use alternatives to current detection methods (Table 2). These simple tests also provide viable methods of medical testing in underdeveloped areas of the world due to their relatively long shelf life, ease of transportation, lack of specialized equipment [36], and affordability [35]. 

#### 2.1.3. Detection of Biologically Relevant Molecules using PDMS-Based Microfluidic Devices

Another approach to detect biomolecules uses traditional PDMS-on-glass microfluidic devices generated by photolithography and PDMS replication. These devices are different from LFSAs and µPADS because PDMS microfluidic devices often rely on an external pressure source to induce the flow of fluids through the device instead of diffusion-driven wicking of fluids across a piece of paper. Microfluidic techniques to detect specific biomolecules for disease identification and progression include the use of microfluidic trapping arrays [42], droplet encapsulation of samples [42,43], on-chip genetic amplification [38], flow-based filtration of samples [39], and capturing biomolecules using antibodies [39,40]. 

PDMS microfluidic devices have been developed to control the mixing of samples with reagents through complex geometries of channels and arrays to produce a measurable output [38,39,40,41]. Other microfluidic devices incorporate droplet generation and trapping to detect biomolecules [42,43]. Analytical outputs for these platforms include fluorescent microscopy [38,40,43], spectroscopy [39], color production and intensity [41], and analysis of individual droplets [42,43]. A device developed by Salim et al. was used to perform genetic amplification on-chip. The microfluidic droplet generator was used to perform quantitative reverse transcription polymerase chain reaction (qRT-PCR) in order to detect overexpression of miRNA 21 as a biomarker for breast cancer in patients by measuring a fluorescent output [38]. Other microfluidic devices utilized antibodies immobilized within the flow channels to capture desired proteins [39,40]. A device developed by Tsai et al. used a sample of whole blood containing filters to remove the red blood cells (RBCs) and capture antibodies to measure the C-reactive protein (CRP) concentration, a useful biomarker for inflammation and necrosis detection. CRP concentration was quantified by binding CRP downstream and measuring this capture by a shift in wavelength of light passing through the device [39]. A similar device used antibody capture of proteins to detect interleukin-2 (IL-2) from lymphocytes. This device passed lymphocyte lysates over a layer of immobilized antibodies and measured the concentration of IL-2 by emitting a fluorescent signal after protein capture [40]. A different approach was used by Qiu et al. to detect CD4, a protein present in T lymphocytes in which low levels indicate HIV. Their device lysed T lymphocytes and measured CD4 using 2 mm beads and a chemiluminescence assay all on chip. This device provided a pseudo single cell analysis by initially lysing cells, reacting CD4 within the lysates to chemiluminesce, and finally calibrating that luminescence to number of CD4+ T lymphocytes [41].

Microfluidic droplet generators have numerous applications due to their ability to rapidly encapsulate single cells or perform biochemical assays with very low limits of detection and high sensitivity. An approach developed by Cui et al. mixed a PSA-spiked HEPES (*N*-2-hydroxyethylpiperazine-*N*-ethane-sulfonicacid) sample with polystyrene beads conjugated to PSA antibody followed by droplet encapsulation to measure PSA concentration by counting the percentage of droplets that had aggregated [42]. Another device developed by Shen et al. measured hydrogen peroxide excreted from cells within a sample. Cells were combined with gold nanoclusters and encapsulated to produce an increasingly intense fluorescent signal as more hydrogen peroxide was excreted. This platform was found to be capable of analyzing 1000 cells per minute [43].

Compared to LFSAs and µPADs, PDMS-based microfluidic devices have greater sensitivity and higher throughput resulting in better limits of detection due to their easily tunable properties (Table 2). Although these devices have not yet been commonly incorporated into the clinic, they are a mainstay in the laboratory and are showing increasing promise as tools for clinical detection purposes. 

#### 2.1.4. Sensors, Chips, and Other Technologies to Detect Biomolecules

Alternate detection methods rely on the use of electronic sensors [47], chips patterned with detection molecules [44,45,46,47,48], devices incorporating nanomaterials [44,45], and imaging platforms [46]. These devices differ greatly in their setup, but all share a similar construction of detection chemicals patterned onto portable metal chips. These devices are developed specifically to analyze patient samples (e.g., blood, urine, and saliva) and to detect biomolecules and are often characterized by their ease of use, all-in-one design, and small sample requirements. Additionally, results from these approaches can be processed quickly (e.g., 20 min), providing optimal conditions for clinical use [46]. 

Many of these microfluidic sensors measure changes in conductance or resistance due to changes in the concentration of biomolecule deposited onto the sensor [44,45,47,48]. Methods to detect these molecules include the use of carbon nanobiosensors (e.g., nanotubes and nanoparticles) [44,45], antibodies [46,47], and aptamers [48]. A device developed by Matta et al. used nanotubes to detect cardiac biomarkers myoglobin, cTn I, and CK-MB by imbedding detection nanotubes within SU-8 on a metallic chip to measure conductance by biomolecule accumulation on the nanotube [44]. Another approach utilized nanotubes to detect PSA as a novel approach for early stage prostate cancer diagnosis. This device measured a change in resistance due to PSA attachment to the nanotubes [45]. Other techniques utilized immobilized antibodies to bind and detect biomarkers. A recent sensor by Castiello et al. detected pancreatic islet hormones insulin, glucagon, and somatostatin at low concentrations in ~20 min by measuring sensor reflectiveness after sample addition [46]. A similar sensor used by Chuang et al. patterned electrodes with antibodies to detect Galectin-1 as a biomarker for bladder cancer by measuring impedance [47]. Other sensing approaches rely on aptamers to provide label-free detection of certain biomolecules. An example of this approach used a chip coated with gold electrodes and RNA aptamers to bind IFNγ for the detection of diseases such as influenza and Johne’s disease [48]. 

Sensing technologies such as these could have clinical uses in the future due to their facile and efficient operation (Table 2). They are also well suited for manufacturing due to their simple designs of metal chips with chemical deposited on top. Compared to other clinical techniques, these sensors typically work faster and are easier to use with necessary equipment. Conversely, depending on the test and apparatus, some of these sensors can be quite expensive leading to many of these devices being used in the laboratory. Additionally, many of these sensing schemes require specialized machinery, which adds to the difficulty in implementing them in the clinic [44,45,46,47,48]. Regardless, these types of technologies have the potential to be integrated into clinical treatment.

### 2.2. Detection of Human Cells

Some diseases are detected by the identification of certain cells within the body such as mutated cells resulting in cancer, increases or decreases in white blood cells indicating infection or immune disease, and red blood cells showing anemia. Microfluidics provide a platform to capture and identify these cells to aid in the early detection of diseases. These devices use blood or tissue samples to identify abnormal cell types or concentrations for clinical diagnosis (Table 3).

#### 2.2.1. Methods to Detect Blood Cells

Several disorders exist that can attack the major constituents of blood including red blood cells, white blood cells, and platelets. Clinical detection of blood cells can alert lab technicians to the presence of these diseases by analyzing red blood cells [51,52], white blood cells [53,54,55], and platelets [56]. Several blood diseases affect the structure or function of RBCs including anemias, polycythemias, and hemolysis. In the clinic, technicians can detect and analyze RBCs using a variety of techniques to aid in the diagnosis of several of these blood diseases. µPADs have proven to be a popular method of detecting RBCs [51,52]. A device by Berry et al. was employed to measure the critical hematological index, a ratio of RBC volume to total blood volume [51,52]. Deviations from the normal range of this index can be used to indicate anemia. Another device by Hegener et al. analyzed RBCs at the single-cell level to illustrate the coagulation of blood samples in patients. This µPAD was used to monitor the migration distance of single RBCs, a metric of coagulation which can aid in the diagnosis of diseases such as hemophilia [51,52].

White blood cells (WBCs), also called leukocytes, protect the body against pathogens and other foreign invaders. Most disorders that affect WBCs result in either an excessive or insufficient number of cells. Detection of WBC concentration can provide essential information of bacterial or viral infection and chronic disease management. An electrochemical sensor developed by Wang et al. was used to measure WBC concentration. This sensor was composed of small electrodes patterned onto a thin layer of gold which produced a voltage that correlated to the number of WBCs present in the sample [53,55]. This approach can help with the diagnosis of infections that affect WBC concentration, such as leucopenia. Alternative approaches have been developed to analyze WBC function, which is affected by several disorders. Schie et al. developed a platform that combined automated imaging microscopy with Raman spectroscopy to enable label-free screening of WBCs [53]. This process enabled the screening of 1000 single cells in less than 20 min and was used to detect cellular abnormalities. Another system developed by Tay et al. examined the dysfunction of neutrophils, a specific subset of WBCs that are linked to type 2 diabetes mellitus pathophysiology (T2DM). This device sorted neutrophils from a small blood sample and then performed phenotypic analysis using NETosis [54,55]. Although this strategy is not capable of high-throughput screening, it does provide important information about WBC function using a single-step device. 

Platelets are a component of blood that function by reacting to blood vessel injuries by forming a clot after binding to human umbilical vein endothelial cells (HUVECs). Any condition that damages HUVECs may have a negative impact on platelets’ ability to clot. Devices have been developed to quantify the adhesive relationship between platelets and damaged HUVECs. One such device uses nanoprobes to label the adhesive molecule E-selectin, which allows for the investigation of platelet adhesion to damaged HUVECs [56]. This device is important as platelet adhesion to oxidatively damaged endothelial cells plays a great role in understanding the pathogenesis of cardiovascular disease. 

#### 2.2.2. Methods to Detect Cancer Cells

Cancer cells exhibit relentless division, which flood the body and form tumors. These cells can spread to other parts of the body in a process known as metastasis. In this process, a circulating tumor cell breaks off from a primary tumor, travels to another location through the vasculature, and forms a secondary tumor. Several studies have been conducted to specifically isolate and detect CTCs, which have been nicely summarized in the following review articles [22,23,24,25,26]. Besides capturing CTCs, early detection and characterization of cancer prior to tumor cell intravasation is imperative to the treatment of an infected patient. The detection of cancer cells has been achieved through use of flow devices [58], while the severity and type of cancer have been assessed through use of microwell arrays and other microfluidic devices [57,59] as well as solid-state micropores [60,61,62]. There exist several approaches to distinguish cancer cells from healthy cells with the most popular being flow cytometry [63]. However, alternatives to flow cytometry have been developed to improve the limit of detection, reduce the cost, and allow for enhanced usability. An optofluidic chip was employed by Pedrol et al. to measure cellular fluorescence to distinguish between cancerous and non-cancerous cells. Cells were tagged by fluorescent antibodies prior to on-chip imaging which utilized embedded optical fibers to detect both EpCAM and HER2 in cell samples [58]. 

Tumor heterogeneity can confound the detection and diagnosis of cancer patients, which has led to the use of high-throughput, single-cell analysis platforms to aid in the determination of cancer malignancy. Tang et al. developed a microwell array device to identify cancer cells by exploiting their altered glucose metabolism. A fluorescence glucose analog (2-NBDG) was used to measure high glucose uptake of cancer cells using high-throughput fluorescence screening to distinguish healthy cells from cancerous cells [57]. This method provided greater insight over cytological analysis by quantifying the single cell response to provide information across the population of cells. Ilyas et al. developed a device to detect bladder cancer cells based on electrical output of cells from a solid-state micropore system [61]. Ali et al. also created a device capable of identifying metastatic and non-metastatic tumor cells using solid-state micropores [62]. Both devices work by converting biophysical properties of the cells such as stiffness, cellular activity, and growth rate into electrical signals as the cells pass through the micropores [61,62]. Similarly, tumor heterogeneity can negatively impact drug efficacy during treatment. The detection of multiple antibodies from a single sample can substantially assist in characterizing drug resistance. Lee et al. used a microfluidic device with multiple chambers capable of multiplexed staining of different antibodies from a single sample. This device exploited the hydrophobicity and hydrophilicity of certain chambers to ensure the antibodies remained in the desired chambers while simultaneously mixing the sample [59]. These types of devices can provide information regarding the personalized nature of cancer, which can in turn lead to more effective treatment. Additional discussion about single cell analysis related to tumor heterogeneity is provided later in this review.

### 2.3. Detection of Bacteria

Bacterial infections affect over two million people on an annual basis within the United States resulting in a need for the early and accurate detection of the specific bacteria infecting a patient [64]. Traditional methods of identifying bacteria require time consuming culturing steps which can sometimes be ineffective. To overcome this limitation, microfluidic devices have been developed to facilitate the rapid detection bacteria (Table 4).

#### 2.3.1. Detection using Blood Samples

One challenge associated with detecting bacteria in the blood is the need to filter out blood cells to identify bacteria, which occur in much lower numbers [66]. Conversely, the entire sample can be lysed which requires additional analysis steps to enhance the detection of bacterial DNA [65,67]. The filtration or lysis steps can occur directly within the device [66] or prior to sample analysis [65,67]. A population approach incorporates an on-chip amplification step to enhance the amount of bacterial DNA needed for detection. For example, a PDMS-on-glass device by Eid and Santiago was used to detect *Listeria monocytogenes* by amplifying the *Listeria* DNA after lysing cells from a whole blood sample. The isolation and amplification of DNA occurred entirely on-chip while sample lysis occurred off-chip. Bacterial identification occurred via quantifying a fluorescent signal proportional to the DNA copy number. The limit of detection for this device was reported as approximately 5000 bacterial cells per milliliter of whole blood [65]. Similarly, a device by Ohlsson et al. was designed to screen blood samples for *Pseudomonas putida* and *E. coli* as a detection scheme for sepsis by amplifying target DNA using polymerase chain reaction (PCR). This device was an all-in-one chip that filtered out red blood cells by acoustophoresis followed by trapping bacteria on polystyrene particles. The bacterial DNA was amplified and detected by a fluorescent signal that increased as DNA multiplied. This device was capable of detecting bacteria as low as 1000 cells per milliliter of blood [66]. An alternative approach was utilized by Choi et al. to facilitate malaria detection in the field. This system operated by lysing the blood sample and loading it into a plastic disc that contained reagents required to amplify the DNA of *Plasmodium falciparum*, the protozoa responsible for malaria. A fluorescent detecting machine read the disc and sent the information to a smart phone to easily interpret results. The entire setup was relatively inexpensive, rechargeable, and easy-to-use, making it an ideal platform for field detection of malaria [67].

#### 2.3.2. Detection using Saliva or Other Samples

Some bacterial detection platforms use samples such as saliva, urine, or even air to identify certain pathogens. These devices use a similar approach to detect bacteria as those described above; however, there is no need for filtering out RBCs and these devices are far less invasive. Horst et al. combined on-chip genetic amplification with lateral flow detection as a novel approach to identify gonorrhea infection. A sample was collected from the infected area and the DNA from the gonorrhea-causing bacterium *Neisseria gonorrhoeae* was amplified to provide a positive or negative result with a limit of detection as low as 10 bacterial cells in a single device [68]. Alternative approaches have been developed to detect bacterial infections around medically implanted or installed equipment [69,70]. A device by Chen et al. sampled the fluid around prosthetic joints to identify seven different bacteria known to cause periprosthetic joint infection (PJI). This method overcame the current method of detection that can take 3–7 days to culture the bacteria within the infection and works by using loop-mediated isothermal amplification (LAMP) of specific genes present in these bacteria all on chip [69]. A device by Hoyos–Nogues et al. detected periodontopathogenic bacteria by sampling the saliva around the dental implant and capturing the bacteria within a device via immobilized antimicrobial peptides. Additionally, these peptides were attached to underlying electrodes, and bacterial detection was measured by resulting changes in resistance with a limit of detection of 10 CFU/mL [70]. Other devices have been designed to identify pathogenic bacteria and bacteria toxins within air samples [71,72]. Bian et al. trapped the bacteria *Vibrio parahemolyticus* within a microfluidic trapping device and performed mass spectrometry to identify the bioaerosols excreted by the bacteria [71]. Jiang et al. developed a device to test air samples by flowing air spiked with bacteria through a microfluidic device coated with LAMP reagents to detect *Staphylococcus aureus* as well as four other common airborne bacteria with a limit of detection of 24 CFU per microfluidic channel for air spiked with *S. aureus* [72]. 

### 2.4. Detection of Viruses

Viral infections present a serious issue to the population. Influenza kills 12,000 to 56,000 Americans annually and hospitalizes an additional 140,000 to 710,000 [73]. Several groups have dedicated their research toward discovering and optimizing methods of detection that can be utilized to quickly and effectively diagnose patients with viral infections including influenza, Zika, and sexually transmitted diseases (Table 4).

#### 2.4.1. Methods to Detect Influenza

Influenza is a highly infectious virus that exists in three different strains. The contagious nature of the disease along with its potentially severe symptoms in patients necessitates sensitive and fast methods of detection. Several microfluidic systems have been fabricated to scan for multiple strains of influenza simultaneously. Fluorescent microscopy coupled with microfluidic channels has been employed to detect multiple types of influenza at the same time [7,8]. Yu et al. used nanorods functionalized with antibodies specific for different strands of the avian influenza virus (AIV) to produce a fluorescent signal to identify the different strands of AIV at once [7]. Wang et al. took advantage of aptamers to detect different strains of influenza. At different conditions, such as changes in pH or temperature, a universal aptamer conjugated to fluorescently tagged, magnetic beads was used to bind and detect different strands of the virus [8]. Both methods screened for different strands of influenza simultaneously and yielded a limit of detection of 3.2 hemagglutinin units (HAU), which is 10 times more sensitive than that of conventional assays. Microfluidic devices have also been developed to overcome the time-consuming steps and excessive reagents currently required for detection. Wu et al. used a nitrocellulose membrane functionalized with antibodies specific to the H1N1 virus to detect influenza A using ELISA. This device utilized gravity and capillary forces to avoid directly pumping the reagents and integrated a smartphone for image capture and analysis [9]. This system included an immunoassay to avoid the nucleic acid amplification necessary in other techniques and included reagent storage and reaction molecules for point-of-care application.

Digital microfluidics (DMF) has proven to be a powerful new platform for immunoassays requiring multiple steps. Wang et al. coupled DMF with surface-enhanced Raman scattering (SERS) to perform rapid, automated, and sensitive detection of AIV. Their platform used a sandwich immunoassay in which magnetic beads coated with antibodies were used to capture H5N1 and detect it using a SERS tag [10]. This method provided a low-cost, highly sensitive, assay to detect influenza other than the standard ELISA. In another example, Prakash et al. used a DMF platform for the polymerase chain reaction (PCR) detection of influenza [74]. The DMF platform was equipped with a nanostructured superhydrophobic surface and yielded a limit of detection of the target influenza virus of less than 10 RNA copies per reaction. These techniques demonstrate the potential of DMF platforms and their ability to detect viruses quickly and sensitively. 

#### 2.4.2. Methods to Detect Zika

Zika virus, which is spread by daytime-active mosquitoes, has historically affected a limited portion of the world in Africa and Asia. However, in the past decade, the virus has spread leading to the Zika virus epidemic in 2016 [75]. This increased scope has led to a need for new methods to rapidly detect the virus. Pardee et al. reported one cost-efficient method to detect clinically relevant concentrations through use of a paper-based sensor [12]. This method involved linking isothermal amplification of Zika RNA to programmable RNA sensors called toehold sensors, which would create a colorimetric change in the presence of Zika RNA. The amplification of viral RNA is an essential component of numerous other devices developed to detect Zika as well [12,13]. Lee et al. combined a lateral flow assay with RNA amplification using RT-LAMP to allow for specific Zika RNA detection down to a single copy level within 35 min [12]. This platform has the potential for point-of-care detection of Zika. Song et al. incorporated a bioluminescent assay involving loop-mediated isothermal amplification (BART-LAMP) with smartphone detection to eliminate the need for an excitation source [13]. This simple, inexpensive, mobile detection platform was capable of rapid and quantitative molecular diagnostics to detect clinically relevant levels of Zika in urine, saliva, and blood samples.

#### 2.4.3. Methods to Detect Sexually Transmitted Diseases

Sexually transmitted diseases (STDs) are infections spread by sexual activity. Viral STDs can be difficult to detect in patient-derived serum samples and are often characterized by a wide variety of symptoms, which range from mild to severe. As such, methods have been developed to identify different viral strands from patient samples. The detection of viruses in blood samples can be difficult due to their dilute concentration requiring sample enrichment steps. Surawathanawises et al. used semi-circular traps composed of porous silica beads and polystyrene to trap viruses and remove RBCs and WBCs [14]. This method was tested using human blood infected with HIV and was capable of efficiently detecting the virus in concentrations of approximately 10^6^ virions per mL. Additionally, multiple reaction chambers can be utilized to test for numerous strands of a virus simultaneously. Qiu et al. fabricated a device using a multi-step chemiluminescence assay to detect herpes. Microbeads were immobilized with one type of antigen and were magnetically guided from one chamber to the next [15]. Use of different antigens enabled the simultaneous screening of five different infections. Additionally, when compared to ELISA, this method had improved sensitivity, making this a possible candidate for future infection screenings.

## 3. Disease Diagnostics

Diseases have traditionally been characterized by histology; however, examining diseased cell colonies or tissues under the microscope can sometimes provide insufficient information about complicated intracellular processes. Additionally, bulk analysis of cells masks crucial information of distinct subpopulations by averaging signals from individual cells, which has led to the use of single cell analysis. This refers to the interpretation of cell-to-cell differences in terms of cellular behavior, morphology, and molecular content such as DNA/RNA and other metabolites. In recent times, microfluidic approaches have been well suited to investigate single cell behavior due to their increased sensitivity, economy of scale and ease of automation. The following sections highlight the different sets of microfluidic devices that have been developed to characterize cellular heterogeneity through phenotypic (cellular behavior) and genotypic (molecular) profiling. Several of these devices have played a critical role in delineating heterogenetic cellular sub-populations by providing phenotypic–genotypic correlations, eventually contributing to an effective understanding of personalized nature of the disease. Beyond profiling, microfluidic devices have been utilized for drug screening where single cell responses to various drugs are assessed to identify the right drug with the right dose.

### 3.1. Cell Behavior

**Figure 2:**
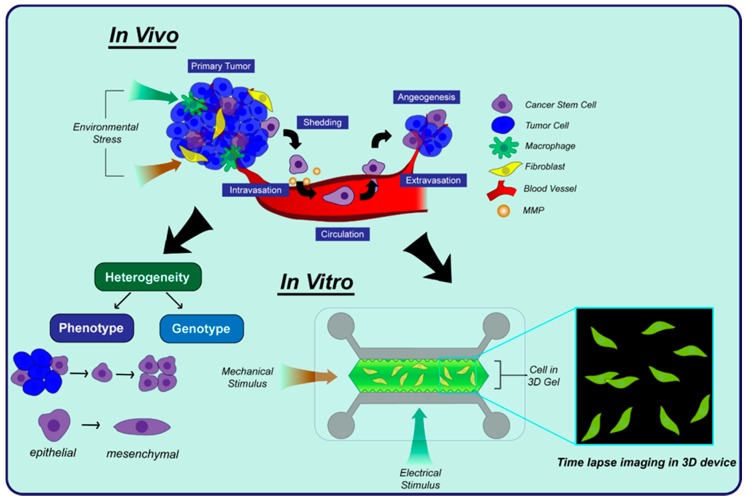
Microfluidic devices to study the cellular microenvironment provide insight on cell behavior. The in vivo microenvironment has cells migrating through the vasculature to form secondary tumors which induce angiogenesis. The heterogeneity of cells can be distinguished by clonal evolution and epithelial to mesenchymal transition. In vitro microfluidic devices can image 3D microenvironments that replicate the ECM to study cell migration and cell-to-cell communication using time-lapse imaging.

The diagnosis of diseases and the success of preclinical studies of a drug is dependent upon a fundamental understanding of cellular behavior. This can depend upon the biophysical properties of cells, the communication between cells, and their response to intracellular gradients [76]. This section focuses on how microfluidic devices have been used to study the behavior of cells. For example, cancer metastasis occurs due to the transition and migration of cells in response to extracellular gradients, which can be mimicked in microfluidic devices (Figure 2). Microfluidic devices can also be used for cell-to-cell communication in organs, tissues, and the tumor microenvironment, which has been shown to accelerate disease progression. A large number of microfluidic devices have been generated to study cellular behavior during cancer metastasis and progression, so for the sake of brevity this review will focus on these devices and their findings. However, many of the devices presented here have applications in a number of diseases beyond cancer.

#### 3.1.1. Single Cell Heterogeneity

The analysis of individual cells yields more precise information about disease progression due to cellular heterogeneity and allows for both end point and dynamic information of intact cells. By only examining cell lysates, the heterogeneity is averaged across the entire population and valuable information about distinct subpopulations is masked or lost entirely. Heterogeneity can result from the cellular microenvironment which consists of biochemical and physical signals including ligands (e.g., growth factors and extracellular matrix (ECM) proteins), mechanical properties, temperature, pH, metabolism, and mechanical/electrical stimuli. These properties affect cellular phenotype and cell behaviors including apoptosis, proliferation, migration, and differentiation [77].

The invasive nature of cells is initiated by epithelial to mesenchymal transition (EMT) which can alter cellular behavior. Li et al. demonstrated single cell heterogeneity and phenotypic modifications during cellular invasion in the presence and absence of an anti-invasion drug. Heterogeneity was distinguished by the expression of E-cadherin and vimentin as epithelial and mesenchymal markers in two different breast cancer cell lines, MCF7 and MDA-MB-231, in a microfluidic chip used to perform single cell studies on EMT [78]. Similarly, Huang et al. utilized time-of-flight secondary ion mass spectrometry (TOF-SIMS) to analyze the heterogeneity across a population of HeLa cells in response to treatment with the anticancer drug cisplatin. They combined TOF-SIMS with a multivariate statistical data analysis tool PCA (principal component analysis) to detect a reduction in cholesterol and fatty acid level after cisplatin treatment, which is a sign of cell apoptosis [79]. Similarly, Anchang et al. characterized a heterogeneous response among individual HeLa cells using mass cytometry time-of-flight (CyTOF). They processed the data in a computational network to optimize combinations of BEZ-235, Dasatinib, and Tofacitinib in the treatment of leukemia by measuring intracellular and surface markers cPARP, Caspase3, and Caspase7. Their analysis found that a combination of two drugs may be the optimal approach for most patients; however, this combination needs to be further optimized [80].

The mechanical properties of cells including stiffness and deformability have been shown to differ from cell-to-cell with certain cell lines exhibiting a greater variation. Mokbel et al. used numerical simulations to characterize single cell deformation in terms of circularity using polyacrylamide beads in a microfluidic device to elucidate how different cells exhibit different mechanical properties. They proposed two types of cell deformation measured by two eigenvalues moment-of-area tensors [81]. Similarly, Deng et al. developed a fully automated high-throughput device to measure cell deformability as a metric of breast cancer cell progression to provide new information about distinctive changes in different breast cancer cell lines including MDA-MB-231 and MCF7. Additionally, their device was capable of identifying cell stiffness change that occurred during EMT [82]. The measurement of cellular electrical properties (e.g., impedance) combined with the quantification of the mechanical properties can provide additional information about the cellular phenotype as another metric to characterize cell heterogeneity. Zhou et al. measured changes in impendence and cellular deformation to identify a set of cellular biomarkers associated with different phenotypes. The high-throughput microfluidic device measured changes of electrical properties in the plasma membrane and cellular deformability through a constriction during deformation stage in MCF7 cells [83]. Luo et al. found that electrical stimulation of pre-osteoblast cells altered cellular differentiation, which was found to be highly heterogeneous. They exposed MC 3T3-E1 (pre-osteoblast) cells to periodical electrical stimulation for short time periods to enhance osteogenic differentiation [84]. This heterogeneous property of clonal expansion can be used for cell screening and drug testing. 

Proteins secreted by cells are used to facilitate cell-to-cell communication which can alter their physio-pathological conditions. Single cell secretomic analysis of protein expression provides a direct measurement of phenotypic properties to characterize cellular heterogeneity [85]. Bai et al. reported a PDMS 3D scaffold-based microarray platform to investigate single cell protein secretion behavior in brain tumor-derived U87 cells. They found a variation in the secretion of IL-8, MCP-1, and IL-6 across the cells under different environmental conditions. This led to the conclusion that protein secretions varied depending on the type of substrate used to culture the cells, which led to an altered behavior that included differences in cell morphology and motility [86]. The single cell protein secretomics was also analyzed in human foreskin fibroblasts to identify the regulation of ECM proteins (e.g., COL1A1, YWHAZ, and MMP) using mass spectrometry (MS). Hu et al. found that all proteins were not secreted equally when foreskin fibroblasts were cultured in a microfluidic system [87]. Li et al. utilized a nanoliter scale oil-air-droplet chip and shotgun proteomics to analyze protein expression in single HeLa cells, which showed improved detection performance by 40% compared to traditional in-tube systems where sample was prepared in tube [88]. As a proof of concept, the microfluidic chip identified 355 proteins when it was used to analyze single mouse oocyte.

#### 3.1.2. 3D Cell Migration

Microfluidic devices are ideal platforms to study cell migration, especially in a 3D environment. Traditional motility studies performed in 2D are not capable of mimicking 3D cellular environment, specifically with respect to invasion. 3D microfluidic systems are more biologically relevant and allow for direct control in simulating physiological conditions coupled with the ability to directly monitor cell movement.

Cellular invasion can be stimulated by exposing cells to external gradients of chemoattractant and growth factors to direct their movement. Truong et al. measured the enhanced 3D invasion in SUM-159, a type of breast carcinoma cells, when the cells were exposed to a gradient of epidermal growth factor (EGF). They utilized a microfluidic platform and identified changes in cell morphology and proliferation when exposed to the EGF gradient. They also found that the SUM-159 cells exhibited enhanced invasiveness when they were co-cultured with cancer-associated fibroblasts (CAFs) [89]. Wong and Searson studied the invasion of the triple negative MDA-MB-231 cell line through ECM and endothelial cells in a microfluidic system which mimicked the invasion and migration behavior during metastasis. They observed slower motility of tumor cells in dense ECM compared to less dense ECM, using migration speed and fluorescence image analysis as migratory parameters [90]. Erdogan et al. found that CAFs promoted the directed migration of DU145 prostate cancer cells by secreting fibronectin that would align with the ECM. By comparing normal fibroblasts with CAFs, they found that the matrix created by CAFs was regulated by α5β1 integrin to enhance the contractility and traction forces of the prostate cancer cells [91]. Chanasakulniyom et al. quantified cell proliferation and migration studies in a microfluidic vertically integrated array using MDA-MB-231 cells. Chemotaxis was measured in the presence of stromal cell derived factor 1α (SDF-1α) in a microarray device, which confirmed that a gradient of SDF-1α enhances invasiveness and migration. They also demonstrated pseudopod formation during cell spreading in response to SDF-1α gradients [92]. Similarly, Hockemeyer et al. investigated cell-to-cell interactions in a 3D microfluidic device using CAFs and MDA-MB-231 cells. MDA-MB-231 cell migrated to a greater extent in the presence of either SDF-1α or CAFs, which suggested that CAFs co-cultured with breast cancer cells are responsible for producing SDF-1α [93]. Kim et al. also observed the enhanced 3D migration of MDA-MB-231 cells in response to combined gradients of SDF-1α and EGF in a microfluidic device which emphasized the importance of the collective role of cytokine and growth factor release in the tumor microenvironment [94]. Blaha et al. investigated the role of cell/matrix interactions during the invasion of MDA-MB-231 cells through collagen gel in a microfluidic device. They found that endothelial cells secreted pro-invasive factors to enhance breast cancer invasiveness significantly [95].

#### 3.1.3. Angiogenesis

Angiogenesis is the cellular process to induce the growth of new blood vessels and is often associated with cancer progression. As such, significant attention has been devoted to studying angiogenesis in 3D environments and how it can be targeted for drug development [96]. Boyden chambers and well plates have traditionally been used to study angiogenesis; however, they are limited in their ability to recapitulate the tumor microenvironment. Conversely, microfluidic-based co-culture of cancer cells and endothelial cells can provide new insight into angiogenesis. Du et al. introduced a droplet microfluidic system to co-culture HUVECs and multiple cancer cell lines (MCF7 cells and two colorectal cancer cell lines, LoVo and HT29) to study the mechanism of angiogenesis. A hypoxic environment was created to induce the secretion of vascular endothelial growth factor (VEGF) to trigger angiogenesis and allow for immunostaining to study tubule formation [97]. They also found that inhibition of angiogenesis occurred in the presence of the anticancer drug Fingolimod by blocking sphingosine-1-phosphate receptor. Nashimoto et al. developed a perfusable vascular network in a microfluidic device to study the 3D co-culture of human lung fibroblasts (hLFs) with HUVECs. The hLFs were found to induce vascular network formation via angiogenic secretion leading to the anastomosis of sprouts from HUVECs into the 3D hLF culture to maintain cellular viability, even in hypoxic condition [98]. Similarly, a 3D angiogenesis microfluidic device reported by Zheng et al. was used to co-culture leukemic cells and bone marrow stromal cells to quantify directional migration and invasion distance in response to pro-angiogenic factors (e.g., VEGF) as well as examining the change in cellular morphology of the endothelial cells [99]. They also quantified angiogenic factor secretions from the leukemic cells and stromal cells. Lin et al. studied the interaction between tumor cells and endothelial cells (ECs) in an integrated microfluidic device, which consisted of three compartments: co-culture, protein detection, and a pretreatment chamber for metabolites. This platform was used to enable cellular crosstalk, monitor cellular phenotype, and detect protein secretion by mass spectrometry. They found that cervical carcinoma cells (CaSki) showed an enhanced resistance to paclitaxel and a higher expression of pro-angiogenic proteins VEGF and PDGF when co-cultured with endothelial cells [100]. 

#### 3.1.4. Cell-to-Cell Communication

Cellular crosstalk is regulated by a signaling pathway induced by paracrine signaling molecules between cells and occurs during development, wound healing, and other physiological processes [101]. Cell-to-cell interactions play an essential role during disease progression, especially in the case of cancer since endothelial cells, stromal cells, and stem cells can all influence the proliferation, migration, invasion, and intracellular signaling of cancer cells. For instance, adipose stem cells (ASCs) growing in close proximity to breast cancer cells can induce phenotypic and genotypic changes in both cell types [102]. There are numerous signaling pathways that are upregulated and downregulated in the cellular microenvironment that can lead to disease progression or remission. Conditioned media and Transwell systems are the methods to study the cell-to-cell interactions; however, they are unable to recapitulate dynamic microenvironment, which has led to the development of a suite of novel microfluidic devices [103].

A microfluidic device developed by Sai et al. facilitated the interrogation of cancer cells exposed to therapeutic drugs in the presence of other cell lines including stromal cells, immune cells, fibroblasts, and endothelial cells. They observed MDA-MB-342 cells sprouting into the surrounding ECM in the presence of CAFs [104]. Rogers et al. reported on the interaction of MDA-MB-231 cells with normal-tissue associated fibroblasts in a 3D co-culture microfluidic system made to induce cellular migration. The fibroblasts were found to create fibril-like branches which assisted in the extravasation of the cancer cells out of the 3D hydrogel [105]. Estrada et al. used a similar approach to co-culture MCF7 cells and normal fibroblasts in alginate droplets to characterize disease progression and drug resistance. The fibroblasts induced collagen deposition during co-culture, which was hypothesized to promote a stiffer matrix to promote tumor cell dissemination. This device was used to observe the release of pro-inflammatory cytokines (e.g., IL-6, CXCL1) during the simultaneous culture of MCF7 and fibroblasts, which corresponded to enhanced tumor aggressiveness and progression [106]. Lewis et al. studied how the co-culture of pulmonary fibroblasts with mouse alveolar epithelial cells, a cancerous alveolar epithelial cell line, influenced tumor–stromal interactions during proliferation, migration, and matrix remodeling. The 3D co-culture system found increased proliferation rates, a greater degree of fibroblast motility, and increased MMP activity [107]. Interestingly, they observed a decrease in MMP activity and migration in the human adenocarcinoma A549 cell line when they were co-cultured with a non-degradable gel. 

Thomsen et al. used micromolding techniques to construct a conical agarose microwell array for the 3D co-culture of two different cell types either in direct contact or at a distance in the hydrogel. This device was used to culture MDA-MB-231 or MCF7 cells with human mesenchymal stromal cells (hMSC) to enhance the 3D proliferation of the cancer cells. They found that exposing the cultured cells to cisplatin and irradiation resulted in undifferentiated and E-cadherin-negative cells [108]. Lu et al. investigated similar cell-to-cell interactions in a body-on-chip system to study the role of adipocytes on insulin secretion by islets of Langerhans. Single islet and adipocytes were loaded in the chip, which resulted in an increase of insulin secretion by the adipocytes. They found that this was due to the release of non-esterified fatty acids, as assessed by electrophoretic immunoassay [109]. Natural killer (NK) cells act by using cell-to-cell interactions to kill target cells, an approach currently under investigation in cancer cell immunotherapy. Ke et al. created an on-chip microenvironment using microwells to preserve the secretions from NK cells to intensify their toxicities toward K562 myelogenous leukemia cells, which showed an increase in apoptotic features when they came into contact with the NK cells [110]. Similarly, immune checkpoint inhibitor (ICI) has emerged as a regulator to enable interactions between cancer cells and immune cells. Tumor-infiltrating lymphocytes (TILs), a type of immune cell, are effective against tumors by responding to ICI [111]. Moore et al. developed a microfluidic system to study TIL effectiveness in destroying tumors by flowing TILs over mouse tumor cells MC38 with or without the presence of an anti-PD-1-ICI and found that ICI-treated TILs mediated elevated tumor killing [112]. 

### 3.2. Analytical Developments in Molecular Profiling

**Figure 3:**
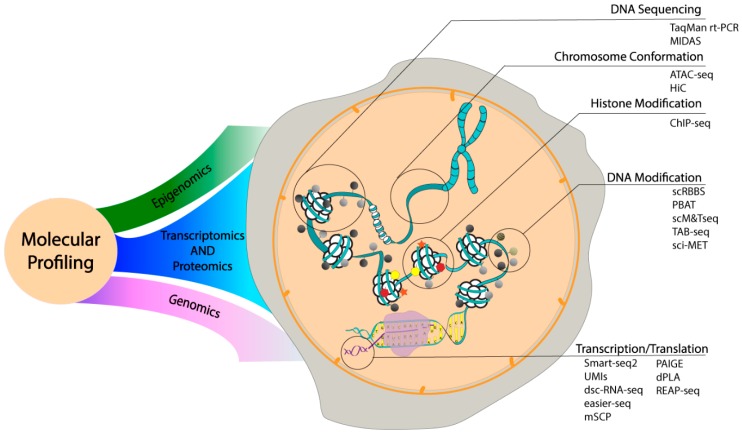
Single cell molecular profiling of DNA, RNA, and proteins. Heterogeneity exists at multiple layers within the cell that can be interrogated using the different technique shown to the right accompanied by an effective microfluidic platform and computational evaluation.

The phenotypic identity of a cell provides valuable information; however, it has become apparent in recent years that cells with similar phenotypic behavior can be heterogeneous in their genetic make-up. Thus, there is a need to understand cellular heterogeneity by examining the distribution and dynamics of its internal molecular components. The current focus of molecular profiling is the large-scale analysis of gene expression using single-cell sequencing. The completion of human genome sequencing in 2001 has provided a foundation of knowledge to build a wide range of high-throughput single cell sequencing tools that provide a comprehensive understanding of cells in diseased and healthy states. To date, several chemical and physical assays capable of single cell molecular profiling have been developed to characterize genetic mutations, intra/extracellular secretomics, dysregulated metabolite content and protein activity [113]. However, these techniques have been hampered by their inability to screen a large number of target molecules coupled with their limited ability to characterize real-time intra or intercellular genetic make-up and kinetic processes due to the complexity imposed by several dynamic parameters. Thus, to address these limitations, microfluidic devices have played a crucial role in capturing and profiling single cells [114,115]. The following section highlights recent microfluidic devices in the field of molecular profiling: single-cell epigenomics, DNA and RNA sequencing, and proteomics (Figure 3).

#### 3.2.1. Single Cell Epigenomics

Epigenetics involves the understanding of physical modifications and conformations of regulatory genetic components, where altering these regulatory systems can enable heritable changes to genetically identical cells. There has been substantial interest in exploiting genetic components (e.g., DNA, histone, chromatin) to identify cellular heterogeneity in seemingly homogeneous populations. Several single cell analytical tools such as DNA folding, DNA methylation, histone modification, chromatin accessibility, and chromosome organization have been developed to measure physical modifications and conformations of these regulatory genetic components [116,117,118,119,120]. Most of these microfluidic devices combine several steps from single cell isolation, barcoding, high-throughput sequencing, and epigenetic profiling to identify patterns across a population of single cells. Lorthongpanich et al. used the commercially available high-throughput C1 Fluidigm chip to recognize differentially methylated regions (DMRs) at multiple loci of early stage embryonic cells from a chimeric mice model [121]. An extension of this work was done by Cheow et al. to perform simultaneous interrogation of gene expression and DNA methylation (sc-GEM) in single human fibroblast cells using the Fluidigm chip [122].

The mapping of histones and chromatin profiling is typically performed using chromatin immunoprecipitation (ChIP) followed by sequencing. Rotem et al. developed a droplet microfluidic device coupled with DNA barcoding to perform next generation sequencing (NGS) called Drop-ChIP-seq [123]. This device involved the encapsulation and lysis of single cells followed by the digestion of chromatin with MNase by fusing the first droplet with a secondary droplet containing oligonucleotides to perform droplet barcoding. These fused droplets were broken down, and Chip-seq was carried out to profile H3 lysine 4 trimethylation (H3K4me3) and dimethylation (H3K4me2) in mixed populations of mouse embryonic stem (ES) cells, embryonic fibroblasts (MEFs), and hematopoietic progenitors (EML cells). Another microfluidic device called the micro­fluidic oscillatory washing–based ChIP-seq (MOWChIP-seq), was developed by Cao et al. to perform high-efficiency ChIP DNA collection while suppressing non-specific adsorption through the use of packed bed beads and effective oscillatory washing [124]. By doing this, the positive and negative loci of H3K4me3 was optimally detected in ~10,000 human lymphoblastoid cell lines. In order to assess regulatory variations in single cell chromatin levels, an assay for transposase-accessible chromatin using sequencing (ATAC-seq) was integrated into a programmable microfluidic platform by Buenrostro et al. [125]. This microfluidic device was used to capture and lyse single human lymphoblastoid cells, followed by ATAC-seq which involved a reaction with the Tn5 transposase enzyme to simultaneously fragment DNA and detect accessible chromatin regions through a process called tagmentation. 

Microfluidic devices have been able to quantify dynamic intra-nuclear states and chromosome organization as shown by Davidson et al. [126] and Jin et al. [127]. In the first work, a novel 3D microfluidic device was constructed to observe real-time intracellular changes to chemical gradients, including nuclear lamina buckling, nuclear volume changes, and chromatin strain during nucleus deformation. In the latter work, a microfluidic device with aperture cell traps was developed to monitor chromosome dynamics in single yeast cells. In combination with high-resolution imaging, this device was able to dynamically analyze chromosome dynamics (e.g., actin and telomere nuclear envelope) in meiotic yeast cells exposed to different stimuli and drugs.

#### 3.2.2. Single Cell Genomics

Owing to the complexity and interdependence of biological systems, diseased states cannot be realistically approached by studying single cells or single genes at a time, which has led to the development of sophisticated platforms to study the activity of multiple genes in parallel. PCR-based DNA and c-DNA arrays have been used to survey the molecular patterns of expression for thousands of genes simultaneously [128,129]. Despite its high-throughput and lower cost of affordability, PCR-DNA microarrays have disadvantages in terms of cross hybridization (10%—low coverage of the single-cell genome) and intensive labor requirement for synthesizing and storing DNA solutions [130,131]. In order to overcome these challenges, microfluidic devices and nanowells have been developed to reduce amplification bias, including reducing the reaction volume and supplementing amplification reactions with single-strand binding proteins to facilitate whole genome sequencing. Zahn et al. developed a microfluidic chip to isolate single cells from triple-negative breast xenograft tumors and conduct nanoliter-volume transposition reactions to generate a whole genome library without sample pre-amplification [132]. A similar ultra-accurate genotyping technique was done by Chu et al. to perform genome sequencing and haplotyping of single human fibroblast cells using SISSOR (single-stranded sequencing using microfluidic reactors) [133]. As a result, this microfluidic device was able to sense single nucleotide polymorphisms (SNPs) by providing accurate and longer haplotypes. 

One of the recent micro-scale advancements was the development of a self-priming microfluidic compartmentalization chip by Zhu et al. to detect PLAU gene expression in A549 lung cancer cells using digital polymerization reactions [134]. Another microwell array method by Li et al. allowed for the rapid generation of uniform emulsion agarose droplets for single molecule analysis using a droplet generator. The sol-gel switching property of agarose was used to form stable beads after digital amplification, thus maintaining the monoclonality of each droplet in downstream processing [135]. The monoclonal beads were subsequently retrieved and subjected to DNA sequencing and FACS analysis. These microwell-based agarose droplets were generated in a high-throughput manner and avoided mammalian cell lysate inhibition of RT-PCR but were unable to use TaqMan probes or cell staining, which precluded a correlation of specific cell types with associated transcriptional targets. Recent work by Eastburn et al. described a novel platform that combined an ultrahigh throughput droplet-based microfluidic technique with high-throughput TaqMan rt-PCR [136]. This platform enabled the specific detection of heterogeneous cellular subpopulations and had a throughput more than two orders of magnitude beyond existing methods. Beyond the primary utilization of droplet microfluidics in single-cell sequencing, advanced multiplexed and massively parallel assays involving cloning and genome sequencing have been done using droplet microfluidics. One such massively parallel sequencing (MPS) technique by Gole et al. involved the use of a microwell displacement amplification system (MIDAS) in nanoliter microwells where single *E. Coli* cells were randomly distributed in nanowells and the genetic material was simultaneously amplified using shortgun sequencing [137]. This device was also used to detect single copy number changes in primary human adult neurons at a 1–2 Mb resolution. A similar short-gun MPS technique was carried out using a micro-array device for cancer genome sequencing in plasma by Chan et al., thus making it a potentially powerful tool for cancer detection and monitoring [138]. 

Genetic mutation studies were also achieved with an aptamer-based microfluidic device, which was developed by Reinholt et al. to capture and isolate genomic DNA (gDNA) from cancer cells [139]. The cancer cells were captured by immobilizing nucleic acid aptamers which were attached to the micropillars of the device. The captured cells were lysed in situ, and the gDNA was collected within a second, smaller micropillar arrays to observe T53 gene mutations. Many label-free optical DNA sequencing techniques have also been employed in recent years. Nagpal et al. developed SERS-based multiplex nanopyramid probes to identify DNA nucleobases with 3D nano focusing [140]. This label-free technique has the potential for developing a high-throughput, block optical sensing method to identify A, T, G, and C content in DNA k-mers.

#### 3.2.3. Single Cell Transcriptomics

mRNA and proteins can provide essential information on the phenotypic identity of a cell. Most single-cell transcriptomic approaches involve the isolation and lysis of single cells followed by reverse transcription (RT) of their mRNA to cDNA and finally amplifying this cDNA through whole transcriptome amplification (WTA). The amplified cDNA is profiled by qRT-PCR, digital-PCR, or RNA-seq [141,142,143]. Despite having several applications in transcriptomics, qRT-PCR and digital-PCR have been overshadowed by RNA-seq due to the ease of use of oligo(dT) primers where polyadenylated [poly(A)] tails of mRNA are universally detected, which prevents a high amplification of rRNA [142]. Recent developments in RNA-seq have included Smart-seq2 and Unique Molecular Identifiers (UMIs) to detect non-polyadenylated mRNA molecules and avoid an amplification bias [144,145]. This progress in RNA-seq techniques and parallel evolution of efficient single-cell sorting systems has led to the emergence of microfluidic-based single cell transcriptomic devices.

Microarrays, nanowells, and droplet microfluidic platforms have gained much attention due to their efficient handling and manipulation of low input RNA samples. Droplet microfluidics have been exploited for cellular barcoding to achieve high-throughput cellular transcriptomics. Droplet barcoding involves tagging the mRNA from each cell with unique barcodes to enable facile library preparation during RT. Two such microfluidic devices are Drop-seq and inDrops. In Drop-seq, Macosko et al. developed an oil–water-based microfluidic platform where individual cells from mouse retinal tissue were encapsulated with microparticles containing barcoding primers [146]. Upon lysis within the droplets, the mRNA from each cell bound to the primers on microparticles, creating beaded STAMPs (single cell transcriptomes attached to microparticles). The barcoded STAMPs were amplified in pools for high-throughput RNA-seq. inDrops is another droplet barcoding device for single-cell RNA sequencing where barcoded hydrogel microspheres (BHMs) were synthesized and combined with a droplet microfluidic platform to index mRNAs thousands of embryonic stem cells for NGS [147]. A similar droplet microfluidic barcoding platform was developed by Rotem et al. to perform high-throughput single cell labeling (Hi-SCL) using barcoded oligo-nucleotides to prime synthesized cDNAs within droplets [148]. A similar droplet-based sequencing technique by Adamson et al. involved coupling single cell RNA-seq with CRISPR-based transcriptional interference (CRISPRi) to mediate genetic inactivation with high efficacy and specificity [149]. A robust cell barcoding strategy was developed to encode the identity of CRISPR-mediated perturbation in an expressed transcript, allowing for the dissection of complex cellular responses in a pooled format.

A droplet microfluidic based RNA-seq method by Kang et al. demonstrated massively parallel profiling of transcriptomes using droplet single-cell RNA sequencing (dscRNA-seq) coupled with a computational tool, demuxlet [150]. This platform enabled multiplexed profiling and computational demultiplexing of transcriptomes from eight different individuals assessing differential expression in terms of SNPs across multiple individuals. Another feasible and effective way of whole-transcriptome profiling was developed by Fu et al. where random N_6_ hexamers were used as primers for RT in emulsion-based amplification of sequence independent evenly transcribed RNA sequencing (easier-seq) [151]. Following RT, a microfluidic device was used to uniformly distribute the cDNA in droplets for individual isothermal amplification reactions. The recent works by George et al. [152] and Shalek et al. [153] demonstrated the capabilities of whole-transcriptome profiling microfluidic platforms to perform dynamic evaluation of single cells as well. In the first work, a splittable single cell microchip was integrated with a high-density antibody array for cytokine protein detection, while the same single cells were sequenced to obtain the genome-wide transcriptome. The other work helped to understand the extent of gene expression variation between seemingly identical cells. Over 1700 primary mouse bone marrow-derived dendritic cells were sequenced for single-cell RNA-seq libraries using a microfluidic device and SMART-seq to characterize different gene modules for regulatory paracrine signaling using temporal heterogeneity profiles. Other than RNA seq, droplet microfluidics have been exploited for single-cell RT-PCR and droplet digital PCR (ddPCR) by Kim et al. [154] and Tian et al. [155]. Non-microfluidic tools have also played a contributory role in the field of single cell transcriptome sequencing. Zhang et al. developed a modular three-part single-cell pipette (mSCP) consisting of a hydro-dynamic trap to isolate single cells in submicroliter volumes, thus enabling single cell PCR analysis and RNA sequencing [156]. This method is a convenient, rapid single-cell isolation technique for isolating single cells for transcriptome analysis. 

#### 3.2.4. Single Cell Proteomics

Although transcriptome profiling provides information about the genetic make-up of cells, proteins are the real products. Transcriptional activity does not give a whole picture of protein abundance and activity due to the complexity involved in the versatile cellular post-translational processes [157,158]. These considerations, in addition to the non-destructive detection (in cell-surface and extracellular secretomic studies) techniques, have facilitated the quantification of protein expression to describe cellular characteristics. Owing to a growing need to study single-cell protein expressions, substantial work has been done to develop microfluidic devices that aid in assessing protein secretion and abundance within cells. 

One of the earliest microfluidic devices by Ma et al. involved the development of a single cell barcode chip (SCBC) that incorporated patterned arrays into microfluidic channels to characterize functional heterogeneity by analyzing the expressions of different proteins from immune cells [159]. Cells were isolated using microvalves and the secreted proteins were captured with DNA-encoded antibody library (DEAL) antibodies. The arrays were stained in a sandwich-ELISA format to detect inflammatory cytokine secretion and effector molecule secretion by single macrophages and tumor-antigen-specific cytotoxic T-lymphocytes. Another tool exploiting DEAL and droplet microfluidics was the REAP-seq assay (RNA expression and protein sequencing assay) employed by Peterson et al. to quantify proteins using barcoded antibodies and >20,000 genes in a single workflow [160]. Additionally, REAP-seq was used to quantify heterogeneity by assessing the effects of a CD27 agonist on human CD8^+^ lymphocytes. Another barcoding technique was developed by Weissleder et al. to analyze hundreds of proteins from minimally invasive fine needle aspirates (FNAs) [161]. In this technique, a magnetically layered microfluidic platform was coupled with an antibody barcoding technique with photocleavable DNA to perform multiplexed protein measurements and analyze single cellular profiles in small amounts of clinical samples. Another approach to quantify proteins in single mammalian cells is droplet digitalization, which was done by Albayrak et al. They employed a digital proximity ligation assay (dPLA) to quantify sensitive and absolute amounts of proteins [162]. The single cells were isolated, lysed, and combined with oligonucleotide-bound antibodies (proximity probes) and connector oligonucleotides. Following a three-step, proximity-hybridization reaction, the oligonucleotide complex became a dsDNA complex after ligation to complete the conversion of target protein to dsDNA, which was amplified and quantified based on fluorescence by ddPCR. 

Shifting the focus from antibody-based protein assays, Abbaspourrad et al. developed a one-pot mix-and read, antibody-free assay called Protein Assay via Induced Gene Expression (PAIGE) using a drop-based mix-and read system [163]. Here, 9E10 hybridoma cells that produced anti-Myc were co-encapsulated and incubated with PAIGE and lysis buffer in ~10,000 drops. Post-incubation, droplets were sorted based on fluorescence intensity and the amount of anti-Myc present in each cell to enable direct quantitation of protein and enzyme in single cells. Microfluidic devices have also played a critical role in understanding protein–protein interactions (PPI). A device by Du et al. utilized single-molecule fluorescence cross-correlation spectroscopy (FCCS) in combination with a microfluidic chip to monitor p53–MDM2 interactions in single cells [164]. Initially, p53 and MDM2 were labeled with green fluorescence protein (GFP) and mCherry followed by mutating three main domains of p53 to study the fluorescence-corelated p53–MDM2 affinity binding in single cells. In addition to quantifying intracellular proteins and PPI, secretory products were successfully quantified using microfluidics by George et al. in a splittable single-cell microchip to integrate a high-density antibody array for cytokine protein detection [152]. A similar secretomic study was accomplished by Jing et al., where an integrated continuous-flow droplet microfluidic assay was developed to effectively detect secretory protease activity of individual viable cells [165].

### 3.3. Drug Screening

**Figure 4:**
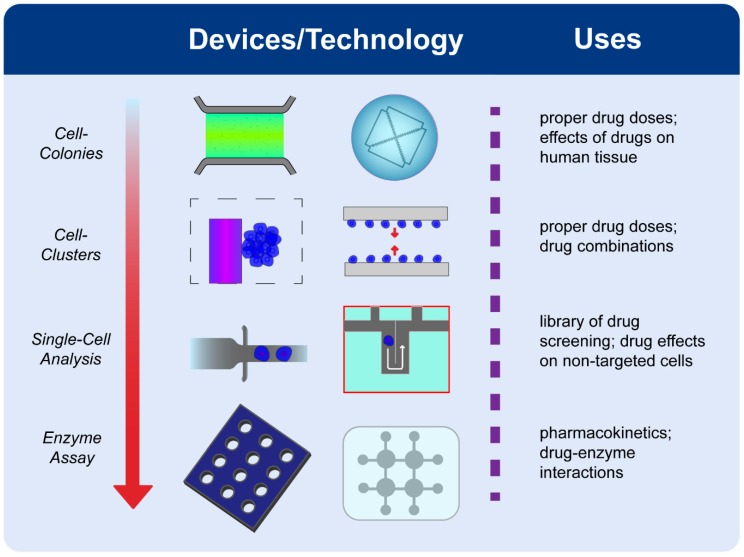
Microfluidic devices used for drug screening and characterization. Drug discovery devices utilize varying levels of cell aggregation, requiring differences in platform design as shown by the examples above. The result is a suite of devices with different geometries and applications to accommodate this differing range of on-chip cell population sizes.

The current drug discovery and development model requires 5 billion USD and 10–15 years on average to obtain one drug to the market [166]. This is due in large part to late-stage failures, which has led to significant efforts to improve both cost and time in the drug development pipeline. On the front end, this involves more efficient segregation of worthy drug candidates, while personalized drug identification and dose selection are needed on the back end. The following section discusses technological advancements to facilitate these improvements ranging from single cell to whole population analyses of single versus combinations of drugs (Figure 4). While these approaches vary in acquired information, required equipment, and clinical adaptability, they all investigate the efficacy of therapeutic drugs, with a strong emphasis on cancer treatment and cardiotoxicity.

#### 3.3.1. Automated High-Throughput Screening

High-throughput screening (HTS) focuses on clinical and/or pharmaceutical industry adaptation; however, conventionally used assays suffer from several weakness. Traditional approaches favor 2D cultures methods in 384-well plates or higher; however, several studies have found that cells respond differently to drugs in 2D environments when compared to in vivo-like 3D environments. Additionally, many systems rely on cell-free, enzyme only assays that cannot recapitulate in vitro findings. Improving these limitations, while still providing automated systems, will allow for accurate drug screening without increasing costs.

Several processes during drug screening can be automated, such as liquid handling, cell culturing, image capture, and image processing [167,168,169]. Two recently developed methods involve traditional 96- and 384-well plates, but the cells are cultured in 3D organoids using a hydrogel scaffold to mimic the cellular microenvironment [167,168]. To further increase biological relevance, patient-derived tumor cells have been utilized during drug screening which leads towards personalized medicine and specific drug selections for individual patients. Jabs et al. used automated image acquisition and processing with confocal microscopy to quantify and differentiate cell death and proliferation [167]. This was accomplished through the comparison of nuclei area of dead and live cells, which was found to be more effective over typical cytoplasmic stains or cellular morphology comparisons. The versatility of this approach was validated with ovarian cancer cells, PDX-derived cells, and lung cancer cells. Boehnke et al. took automation one step further by incorporating automated liquid handling for cell culture and drug administration to increase reproducibility between screens [168]. They utilized a cell seeding robotic platform to culture 3D organoids from single cells derived from colon cancer patients. One challenge associated with 3D culturing is that different types of cells require specific culturing conditions, which limits the global approach of some of these devices. As such, many current studies focus on only specific diseases, and more research is necessary to make these techniques broadly applicable for more diseases.

Another limitation with traditional HTS methods is the requirement to label the cells with fluorescent markers. Quantitative phase imaging has become popular to eliminate the need for invasive approaches; however, the scale has not been large enough for clinical drug screening. To overcome this limitation, Huang et al. developed a high-speed live-cell interferometry technique to investigate tumor cell drug sensitivity. This technique measured tumor therapy response through optical cell biomass measurements and was validated by comparing BRAFi-sensitive and BRAFi-resistant melanoma cells treated with a BRAF inhibitor (BRAFi), blocking the BRAF gene which is responsible for producing B-raf and causes approximately 50% of melanomas when mutated. The BRAFi-sensitive cell line exhibited a significant reduction in cell growth compared to the resistant line. While this method allowed for an increase in throughput, it suffered from the use of 2D cell cultures, limiting its physiological relevance [169]. The new methods discussed here focus on increasing either physiological relevance or throughput. While they show promise for automated drug-screening and personalized medicine, an approach encompassing both factors would be ideal for clinical integration and disease diagnostics.

#### 3.3.2. Microfluidic Devices to Study Cytotoxic Effects and Pharmacokinetics

In contrast to traditional methods, PDMS-on-glass microfluidic devices require fewer reagents and fewer cells, have decreased assay times, and have better single cell resolution. These devices present a much broader functionality when compared to the automated assays, allowing for the investigation of several key factors beyond whether a drug is cytotoxic. Several devices have been developed to interrogate drug efficacy in several diseases [170,171]. For the sake of brevity, this section will mainly focus on devices that have been used for cancer treatment including those that have been developed to uncover direct cytotoxic effects [172,173,174], effects on cell growth [175], and pharmacokinetics [176,177]. Devices studying cytotoxic effects are more applicable to both personalized medicine and drug screening, while devices used to examine cell growth and pharmacokinetics provide a greater understanding of drug mechanics inside the cell. 

Examining cytotoxic effects is of primary interest to determine drug effectiveness and is quantified with on-chip fluorescent staining of cells. These devices focus on creating more accurate physiological models, increasing the throughput of drug concentrations studies using gradient generators, or allowing for the generation and isolation of 3D spheroids using droplet generators. Zhao et al. cultured 3D cell spheroids attached to the sidewall of a PDMS device in a droplet array [172]. This droplet positioning facilitated drug treatment and cell staining, which was performed by taking advantage of capillary forces. The device was used to study the effect of doxorubicin on HepG2 cells, which revealed a higher viability when compared to 2D 96-well plate studies. Further, most of the dead cells in the spheroid were located on the outer surface, while the core exhibited a higher viability. Li et al. exposed cells to a stream of sodium hydroxide and pyrogallol to create a modulated hypoxic environment for oxygen-dependent drug testing, as most solid tumors have extracellular oxygen concentrations well below atmospheric conditions [173]. This approach revealed oxygen concentration has a drastic influence on drug sensitivity. After exposure to two anticancer drugs, TPZ and cisplatin, A549 cells showed opposite trends in viability as oxygen concentration decreased; lower oxygen concentration caused TPZ to be less effective at killing cells, while cisplatin became more effective. Sun et al. developed a gradient generator capable of exposing cells to two-drug combinations [174]. To prove the functionality of this device, an optimal concentration of paclitaxel and cisplatin against A549 cells was determined. 

Cell growth and pharmacokinetics play an important role in evaluating drug efficacy by providing details about absorption, distribution, metabolism, and excretion in cells. Stevens et al. developed a microfluidic approach to measure cell growth using a cantilever-based mass sensor to measure the buoyant mass of single cells [175]. Continuous measurements were taken every 15 min to determine if the cell would continue to proliferate or undergo cell cycle arrest. Novel devices to characterize pharmacokinetics incorporate label-free quantification methods by either modified matrix-assisted laser desorption ionization mass spectrometry imaging (MALDI-MSI) [176] or by SERS [177]. LaBonia et al. investigated drug combinations on colon cancer cell spheroids cultured inside of a reusable microfluidic device using MALDI-MSI. Penetration of the drugs to the center of the spheroid was observed along with a higher metabolism of the drug in the outer region of the spheroid. SERS has the advantage of allowing real time imaging on-chip, while the cell culture must be removed from the device for the MALDI-MSI approach. Fei et al. exploited this advantage of SERS to examine the uptake and expulsion speed of 6-mercaptopurine and methimazole in HeLa cells, noting that it takes only 4 min for the drugs to diffuse into the cells but 36 hours for the drugs to be excreted.

Droplet arrays have applications in both enzymatic and cell-based assays for drug-screening. These devices take advantage of hydrophobic and hydrophilic materials to control the placement of microliter amounts of fluids to create large arrays of pL-sized micro-droplets. These devices do not require external pumps or tubing, so they are easy to control and use. The enzymatic assays mainly focus on identifying specific inhibitors and use arrays with hydrophobic coatings to create the small droplets [178,179]. These small droplets function as single microreactors that contain a substrate and an inhibitor capable of low limit of detection enzyme kinetics. Chen et al. used fluorescence as an analytical output to measure the effectiveness of histone acetyltransferase inhibitors on *Plasmodium falciparum* enzymes [178]. Conversely, Cho et al. relied on secondary ion mass spectroscopy technique to measure protein kinase activity after exposure to inhibitors [179]. Popova et al. fabricated a device for a cell-based droplet assay creating small hydrophilic regions on a hydrophobic surface to facilitate the spontaneous formation of microdroplets when fluid was applied to the surface [180]. Cells were cultured in these microdroplets and placed together with another array of microdroplets containing anticancer drugs. This allowed for a direct and easy screening of drug treatment effectiveness on cells. As a proof of concept, HeLa and HEK293 cells’ sensitivity to doxorubicin was studied, showing equivalent results to standard well-plate studies.

Monitoring the side effects of chemical therapeutics is also an important part of the drug development process. Cardiotoxicity is responsible for approximately one-third of drug failures, highlighting the need to develop devices to better predict the cardiotoxicity of drugs [181]. Espulgar et al. developed a device that took advantage of centrifugal forces to capture and isolate single neonatal rat cardiomyocytes [182]. They used this device to study drug effects on single cells by quantifying beat rate metrics determined by motion tracking software with brightfield microscopy. Mathur et al. used a similar approach to quantify drug effects, but they used human-induced pluripotent stem cells (hiPSC) derived from cardiomyocytes to build a more relevant physiological model [183]. The cardiomyocytes were cultured in a chamber with adjacent media channels that mimicked human tissue and vasculature structure. While different in approach, both devices demonstrated the ability to examine drug-induced cardiotoxicity effects comparable to clinical observations. One limitation to these approaches is their low-throughput nature, as they only had the ability to test the effects of one drug at a time. While this is advantageous for small scale studies, large library screenings of cardiotoxicity cannot be feasibly performed using these devices. In order for the drug development pipeline to take full advantage of these microfluidic technologies, an increased understanding of drug–cell interactions found using microfluidic devices must be scaled up into higher-throughput, automated systems. 

## 4. Conclusions

While many detection platforms observed within the current literature have been used exclusively within a research setting, many of these devices including LFSAs, µPADs, and PDMS-on-glass devices exhibit significant promise to be used within the clinical setting in the near future. These devices are capable of providing clinically relevant detection limits and are designed to produce fast results with both attributes superior to current approaches. The microfluidic approaches are also affordable and easy to use, making them candidates for use at home. Recent studies using microfluidic devices have increased the fundamental understanding of several aspects of disease progression including proliferation, migration, and angiogenesis. The reviewed microfluidic platforms can recapitulate the 3D cellular microenvironment to investigate cell-to-cell communications, which can lead to enhanced disease progression. Additionally, the single cell capabilities of many of these platforms have provided new insight into cellular heterogeneity to identify genes and proteins of interest that are mutated or dysregulated in many diseases, with many studies focusing on cancer. Consequently, researchers have developed easier and more efficient ways to sequence single cell genetic information through molecular profiling, such as single-cell epigenomics and DNA/RNA sequencing, to understand in-depth cellular heterogeneity and cell-to-cell interactions and to prescribe better medications. These approaches have also been used and are still in development to rapidly test multiple drugs in response to those observed behaviors. It is anticipated that microfluidic platforms with detection and analysis systems will be able to optimize doses of drugs in clinics to personalized medicine for heterogeneous diseases.

## Figures and Tables

**Table 1:** Device methods and technical specifications for biomarker detection.

Biomolecule(s) Detected	Type of Device	Method of Detection	Results Readout	Limit of Detection	Total Time (min)	Sample Type	Quantitative	Ref.
Myoglobin	LFSA	Antibodies with PtNPs, H_2_O_2_ reaction forming O_2_ gas	Increase in pressure	2.9 ng/mL	20	Dilute serum	yes	[31]
Tetrodotoxin	LFSA	Gold nanoflower conjugated antibodies, quantum dots	Quantum dot fluorescence	0.2 ng/mL	8	TTX spiked PBS	yes	[32]
Vaspin	LFSA	Complimentary aptamers and AuNPs	Colorimetric intensity	0.137 nM in buffer 0.105 nM in serum	5	Vaspin spiked buffer and serum	yes	[30]
Extracellular vesicles	LFSA	Colloidal gold, carbon black, magnetic nanoparticle conjugated antibodies	Colorimetric intensity	3.4 × 10^6^ EVs/μL	15	Human plasma	yes	[33]
Myeloperoxidase	LFSA	AuNP conjugated antibodies	Colorimetric intensity	250 ng/mL	15	Human sputum	yes	[29]
Glucose, nitrites, and protein	μPAD	Chemical reactions with biomolecules and paper actuator	Colorimetric	n.a.	12	Artificial saliva	no	[34]
Phenylalanine	μPAD	Phe reaction forming NH_3_ and pH change	Colorimetric intensity	20 μM	20	Urine	yes	[35]
Glucose, pH, and protein	μPAD	Enzymes and chromogenic agents	Colorimetric	2 mM 0.6 mg/mL	5	Artificial Urine	yes	[36]
Lactate	μPAD	Electrochemilumin-escence reaction	ECL intensity	0.035 mM	n.a.	Saliva	yes	[37]
miRNA 21	PDMS	Molecular beacon probe	Fluorescence	n.a.	30	Blood	yes	[38]
CRP	PDMS	Capture antibodies	Spectrometry shift	3.2 ng/mL	60	Blood	yes	[39]
IL-2	PDMS	Capture antibodies	Fluorescence	50 pg/mL	30	Blood	yes	[40]
CD4	PDMS	2 mm beads and chemiluminescence assay	Chemiluminescence	75 cells/μL	45	Blood	yes	[41]
PSA	PDMS	Poly styrene beads with antibodies	Droplet counting	3.67 pM	45	Spiked HEPES	yes	[42]
H_2_O_2_	PDMS	Horseradish peroxidase-Au nanoclusters and droplets	Fluorescence	200 amol	90	Cell cultures	yes	[43]
Myoglobin, cTn I, CK-MB	Chip	Carbon nanotubes and antibodies	Conductance	6 fg/mL 50 fg/mL 20 fg/mL	<1	Spiked PBS	yes	[44]
PSA	Chip	Carbon nanotubes and antibodies	Resistance	1.18 ng/mL	120	PSA solution	yes	[45]
Insulin, glucagon, and somatostatin	Chip	Antibodies	Surface plasmon resonance	1 nM 4 nM 246 nM	~20	Spiked solution	yes	[46]
Galectin-1	Chip	Alumina nanoparticles, antibodies	Impedance	7.8 μg/mL	30	T24 cell lysates	yes	[47]
IFN-γ	Chip	RNA aptamer on gold electrode array	Impedance	11.56 pM	<35	Spiked solutions	yes	[48]

LFSA: lateral flow strip assays; µPAD: microfluidic paper-based analytical devices; n.a.: not available.

**Table 2:** Advantages and disadvantages of devices used to detect biomolecules.

Type of Device	Advantages	Disadvantages	Ref.
LFSA	Easy to use at home or in clinic, inexpensive, quick results	Some currently have poor limit of detection, most in research setting only, majority not quantitative	[29,30,31,32,33]
μPAD	Easy to use, cheapest type of device, quick results, easy storage and disposal, can be quantitative, require small sample	Some currently have poor limit of detection, must in research setting only, some required non-ambient conditions	[34,35,36,37]
PDMS	Highly sensitive, easily controllable, relatively inexpensive, requires small amount of sample, high throughput	Requires special training and equipment for use, almost no use in clinics currently	[38,39,40,41,42,43]
Chip	Easy to use, quick results, requires small amount of sample, sensitive limits of detection, easy to manufacture	Many require special equipment, can be expensive depending on test	[44,45,46,47,48]

**Table 3:** Methods of detection and parameters for human cell detection devices.

Cell(s) Detected	Type of Device	Method of Detection	Results Readout	Limit of Detection	Total Time (min)	Sample Type	Quantitative	Ref.
RBCs	LFSA	RBC migration distance to determine coagulation	RBC migration distance	n.a.	4	Whole blood	no	[51]
RBCs	μPAD	Directed flow of cells to determine hematocrit	Blood travel distance	n.a.	30	Whole blood	yes	[52]
WBCs	HTS-RS	Combined automated imaging microscopy with Raman spectroscopy	Raman spectra	n.a.	20	Extracted WBCs	yes	[53]
WBCs	Chip	Small electrodes patterned onto a thin layer of gold	Voltage	195 cells/µL	20	WBCs in 1 mM ferricyanide/ferrocyanide	yes	[55]
WBCs	PDMS	Chemotaxis and NETosis for neutrophil sorting and phenotyping	Fluorescence	n.a.	120	Whole blood	yes	[54]
Platelet	ECL	Adhesion molecule E-selectin as marker site on damaged HUVEC	ECL intensity	1 platelet	12	Platelet-rich plasma	yes	[56]
Cancer Cell	Microwell	Fluorescent glucose analog (2-NBDG) to detect high glucose uptake	Fluorescence	n.a.	10	PE sample	yes	[57]
Cancer Cell	PDMS	Fluorescence-tagged antibodies	Colori-metric	10^6^ cells/mL	1.5	Serum sample	yes	[58]
Cancer Cell	PDMS	Six different antibodies for staining	Staining	n.a.	140	Serum sample	no	[59]

n.a.: not available.

**Table 4:** Target pathogens, methods of detection, and assay specifications for bacteria and virus detection devices.

Pathogen(s) Detected	Type of Device	Method of Detection	Results Readout	LOD	Total Time (min)	Sample Type	Quant.	Ref.
*Listeria monocytogenes*	PDMS	Isotachophoresis purification and recombinase polymerase amplification	Fluorescence	5000 cells/mL	<50	Spiked Blood	yes	[65]
*Pseudomonas putida* and *Escherichia coli*	PDMS	Acoustic RBC separation and PCR	Fluorescence	1000 cells/mL	n.a.	Blood	no	[66]
*Plasmodium falciparum*	Chip	LAMP	Fluorescence	0.6 cells/μL	<40	Blood	yes	[67]
*Neisseria gonorrhoeae*	μPAD	tHDA	Colorimetric	10 cells	60	Genital swabs	no	[68]
*Acinetobacter baumannii*, CNS, *Escherichia coli*, *Staphylococcus aureus*, and MRSA	PDMS	LAMP and ethidium monoazide (EMA)	Fluorescence	~1 CFU	~60	Spiked solution	no	[69]
*Streptococcus sanguinis*	Chip	Immobilized antimicrobial peptides on electrodes	Impedance	10 CFU/mL	~60	Artificial saliva	yes	[70]
*Vibrio parahaemolyticus*	PDMS	Cell trapping	Mass spectrometry	15 CFU	20	Spiked air	yes	[71]
*Staphylococcus aureus*, *Escherichia coli*, *Pseudomonas aeruginosa*, *Citrobacter koseri*, and *Klebsiella pneumonia*	PDMS	LAMP	Fluorescence	24 cells	<60	Airborne bacterial lysates	yes	[72]
H5N2 avian influenza virus	Chip	ZnO nanorods functionalized with antibodies	Fluorescence	3.6 × 10^3^ EID_50_/mL	25	Dilute sample	yes	[7]
H1N1, H3N2, and influenza B	PDMS	Universal aptamer conjugated to magnetic beads	Fluorescence	3.2 HAU	20	Purified RNA	no	[8]
H1N1 and influenza A	Chip	Nitrocellulose membrane functionalized with antibodies for ELISA	Colorimetric	32 × 10^−4^ HA	20	Lysed sample	yes	[9]
H5N1 avian influenza virus	DMF	SERS-based immunoassay	Absorbance	74 pg/mL	50	Human serum	yes	[10]
Zika virus and HIV	Phone	Bioluminescent assay with BART-LAMP	Luminescence	5 PFU	45	Blood, saliva, urine	yes	[11]
Zika virus	μPAD	Toehold sensor linked to RNA amplification	Colorimetric	3 fM	30	RNA in serum	yes	[12]
Zika virus	LFSA	Incorporation of RT-LAMP	Colorimetric	One copy of RNA	35	Blood	yes	[13]
HIV	PDMS	Traps from porous silica beads and polystyrene	Fluorescence	n.a.	60	Blood plasma	yes	[14]
TOX, RUB, CMV, HSV-1, and HSV-2 herpes	Chip	Chemiluminescence immunoassay	Luminescence	32-fold dilution	30	Serum sample	yes	[15]

n.a.: not available; quant.: quantitative; LOD: limit of detection.

## Abbreviations

PSA	Prostate Specific Antigen
PDMS	Polydimethylsiloxane
CTCs	Circulating Tumor Cells
HIV	Human Immunodeficiency Virus
AIDS	Acquired Immune Deficiency Syndrome
LFSAs	Lateral Flow Strip Assays
TTX	Tetrodotoxin
ELISA	Enzyme Linked Immunosorbent Assay
PKU	Phenylketonuria
qRT-PCR	Quantitative Reverse Transcription Polymerase Chain Reaction
RBCs	Red Blood Cells
CRP	C-reactive protein
IL-2	Interleukin-2
HEPES	N-2-hydroxyethhylpiperazine-N-ethane-sulfonicacid
cTn I	Cardiac Troponine I
CK-MB	Creatine Kinase-MB
IFNγ	Interferon γ
WBCs	White Blood Cells
T2DM	Type 2 Diabetes Mellitus
HUVECs	Human Umbilical Vein Endothelial Cells
EpCAM	Epithelial Cell Adhesion Molecule
2-NBDG	2-Deoxy-2-[(7-nitro-2,1,3-benzoxadiazol-4-yl)amino]-d-glucose
ECL	Electrochemiluminescence
CD4	Cluster of Differentiation 4
EVs	Extracellular Vesicles
PtNPs	Platinum Nanoparticles
AuNPs	Gold Nanoparticles
Phe	Phenylalanine
PBS	Phosphate Buffered Saline
PCR	Polymerase Chain Reaction
PJI	Periprosthetic Joint Infection
LAMP	Loop Mediated Isothermal Amplification
CFU	Colony Forming Unit
AIV	Avian Influenza Virus
HAU	Hemagglutinin Unit
SERS	Surface-Enhanced Raman Scattering
DMF	Digital Microfluidics
BART	Bioluminescent Real Time Reporter
RT-LAMP	Reverse Transcription Loop Mediated Isothermal Amplification
STDs	Sexually Transmitted Diseases
tHDA	Thermophilic Helicase-Dependent Amplification
PFU	Plaque Forming Unit
EMA	Ethidium Monoazide
USD	United States Dollar
HTS	High-Throughput Screening
MALDI-MSI	Matrix-Assisted Laser Desorption Ionization-Mass Spectrometry Imaging
hiPSC	Human Induced Pluripotent Stem Cells
ECM	Extracellular Matrix
EMT	Epithelial to Mesenchymal Transition
TOF-SIMS	Time-of-Flight Secondary Ion Mass Spectrometry
PCA	Principal Component Analysis
CyTOF	Cytometry Time of Flight
EGF	Epidermal Growth Factor
CAFs	Cancer-Associated Fibroblasts
SDF-1α	Stromal Cell-Derived Factor 1α
VEGF	Vascular Epidermal Growth Factor
hLFs	Human Lung Fibroblasts
ECs	Endothelial Cells
PDGF	Platelet-Derived Growth Factor
ASCs	Adipose Stem Cells
MMP	Matrix Metalloproteinase
hMSC	Human Mesenchymal Stromal Cells
NK	Natural Killer
ICI	Immune Checkpoint Inhibitor
TILs	Tumor-Infiltrating Lymphocytes
RT	Reverse Transcription
DMRs	Differentially Methylated Regions
sc-GEM	Single-Cell Analysis of Genotype, Expression, and Methylation
ChIP	Chromatin Immunoprecipitation
NGS	Next Generation Sequencing
H3K4me3	H3 Lysine 4 Trimethylation
H3K4me2	H3 Lysine 4 Dimethylation
ES	Embryonic Stem
MEFs	Mouse Embryonic Fibroblasts
MOWChIP-seq	Microfluidic Oscillatory Washing-Based ChIP-seq
ATAC-seq	Assay for Transposase-Accessible Chromatin Sequencing
SISSOR	Single-Stranded Sequencing Using Microfluidic Reactors
SNPs	Single Nucleotide Polymorphisms
MPS	Massively Parallel Sequencing
MIDAS	Microwell Displacement Amplification System
gDNA	Genomic DNA
WTA	Whole Transcriptome Amplification
UMIs	Unique Molecular Identifiers
STAMPs	Single Cell Transcriptomes Attached to Microparticles
BHMs	Barcoded Hydrogel Microspheres
Hi-SCL	High-Throughput Single Cell Labeling
dscRNA-seq	Droplet Single-Cell RNA Sequencing
easier-seq	Emulsion-Based Amplification of Sequence Independent Evenly Transcribed RNA-seq
ddPC	Droplet Digital PCR
mSCP	Modular Three-Part Single-Cell Pipette
SCBC	Single Cell Barcode Chip
DEAL	DNA-Encoded Antibody Library
REAP	RNA Expression and Protein
FNAs	Fine Needle Aspirates
dPLA	Digital Proximity Ligation Assay
PAIGE	Protein Assay via Induced Gene Expression
PPI	Protein-Protein Interactions
FCCS	Fluorescence Cross-Correlation Spectroscopy
GFP	Green Fluorescence Protein
μPAD	Microfluidic Paper-Based Analytical Devices
LOD	Limit of Detection
